# Differences in Heart Rate Variability in the Frequency Domain between Different Groups of Patients

**DOI:** 10.3390/medicina60060900

**Published:** 2024-05-29

**Authors:** Artūrs Garbilis, Jānis Mednieks

**Affiliations:** 1 Department of Residency, Rīga Stradiņš University, Dzirciema 16, LV-1007 Rīga, Latvia; 2 Department of Neurology and Neurosurgery, Rīga Stradiņš University, Pilsoņu 13, LV-1002 Rīga, Latvia; janis.mednieks@gmail.com

**Keywords:** heart rate variability, frequency domain, orthostatic intolerance, neurodegenerative disorder

## Abstract

*Background and Objectives*: Heart rate variability (HRV) is defined as a physiological variation in duration between sinus beats. The aim of this study was to research and analyze the HRV between various groups of patients. *Materials and Methods*: A retrospective study was conducted in an outpatient setting. Patients who had undergone a tilt-table test were selected for this study and were divided into three groups based on their self-reported health anamnesis: group 1 (*n* = 84, mean age 45.8 ± 17.8) consisted of patients with no known orthostatic intolerance or neurodegenerative disease, group 2 consisted of patients with a known or suspected orthostatic intolerance (*n* = 50, mean age 46.5 ± 18.6), and group 3 consisted of patients with a known or suspected neurodegenerative disorder (*n* = 29, mean age 55.6 ± 20.4). During the tilt-table test, HRV frequency-domain parameters—normalized low frequency (LFnu) and high frequency (HFnu), absolute powers—absolute low frequency (LF-RRI), absolute high frequency (HF-RRI), and LF/HF ratio—were recorded during 5 min rest in the supine position. *Results*: Group 1 had a reduced LFnu at 52.93% (SD: 18.00) compared to group 2 at 58.57% (18.06) and group 3 at 61.80% (SD: 17.74), and group 1 had increased HFnu: group 1—47.08% (SD: 17.97), group 2—41.41% (SD: 18.03), and group 3—38.16% (SD: 14.7). LFnu and HFnu differences were statistically significant (*p <* 0.05). LF-RRI was reported as follows: group 1—531.32 ms^2^ (SD: 578.57), group 2—346.2 ms^2^ (SD: 447.96), and group 3—143.21 ms^2^ (SD: 166.96). HF-RRI was reported as follows: group 1—835.87 ms^2^ (SD: 1625.42), group 2—297.46 ms^2^ (SD: 507.15), and group 3—70.83 ms^2^ (SD: 75.67). LF-RRI and HF-RRI comparisons between groups were statistically significant (*p* < 0.001). LF/HF ratios were reported as follows: group 1—1.91 (SD: 2.29), group 2—2.43 (SD: 2.33), and group 3–2.54 (SD: 2.17). LF/HF ratio comparisons between groups were statistically significant at *p* < 0.05. *Conclusions*: This study shows that patients with known or suspected orthostatic intolerance and neurodegenerative disorders have reduced HRV, possibly caused by reduced parasympathetic modulation. HRV in patients with known or suspected neurodegenerative disorders is reduced more severely than in patients with orthostatic disorders. Other studies in HRV have indicated a possible increase of risk in cardiovascular disorders in patients with reduced HRV, and therefore, HRV analysis could be a potential clinical diagnostic tool. However, the lack of universally agreed upon methodology, reference values, and possible external and internal factor influence hinders the introduction of HRV examinations into wider clinical practice.

## 1. Introduction

The human autonomic nervous system plays an important role in the optimization of the cardiovascular system [1,2]. The system is predominantly an efferent system that transmits impulses from the central nervous system (CNS) to peripheral organs. Its nerve fibers can affect heart rate, the constriction and dilation of smooth muscle cells in various organs, and glandular secretions [2]. The autonomic nervous system is usually divided into the sympathetic and parasympathetic nervous systems [2,3].

Heart rate variability is defined as a physiological variation in duration between sinus beats. It allows for the cardiovascular system to respond to various psychological and physical stress factors. Heart rate variability is influenced by the combined activity of the sympathetic and parasympathetic nervous systems and can serve as a measurable indicator of cardiovascular integrity and prognosis. Heart rate variability can be analyzed within two domains: time-domain measures and frequency-domain measures [4,5,6].

A time-domain analysis assesses the difference between normal R-R intervals (NN), excluding ectopic beats. 

Frequency-domain measures of heart rate variability include methods that allow for a distinction between high-frequency (HF) and low-frequency (LF) components. HF components generally range between 0.14 and 0.40 Hz and reflect the activity of the PNS, while LF components range between 0.04 and 0.15 and reflect the activity of both the sympathetic nervous system and parasympathetic nervous system. There is also a very low-frequency (VLF) component that ranges from 0.003 Hz to 0.04 Hz. This reflects a combination of factors, which include not only the sympathetic nervous system but also input from thermoreceptors, chemoreceptors, the renin-angiotensin system, and others [4,5,6].

Multiple devices are available on the market to measure heart variability. The European Society of Cardiology and the North American Society of Pacing and Electrophysiology created the Task Force’s recommendations. The measurements can be either long-term (24 h) or short-term recordings (5 min). The gold standard is multi-lead ECG systems (Holter monitoring); these can provide a more comprehensive HRV analysis, which includes many responses to various environmental factors. The main drawback of this is the required equipment and the burden on both the patients and technicians. Alternative methods involve single-lead ECG or photoplethysmography, pulse oximetry, heart auscultation (sound), and blood pressure monitors. While heart auscultation and pulse oximetry are rarely used in HRT measurement, modern advances in adhesive single-lead ECG and smartphone apps with attachable wrist or chest straps have made HRV analyses more available to the public and at a lower cost [7,8,9].

The heart rate variability phenomenon has been actively researched in the cardiology field, but research in patients with neurological disorders is limited. The currently available data show that a low HRV indicates a higher risk of cardiac arrhythmias and cardiac deaths. Since neurological impairments can also affect the cardiovascular system via a damaged or impaired autonomic nervous system, the goal of this research was to increase the awareness of cardiovascular health in patients with neurological conditions [10,11].

## 2. Materials and Methods

### 2.1. Patient Data Collection

This retrospective study was conducted in “VCA Polyclinic Pļavnieki”, an outpatient clinic located in Riga, Latvia. The study utilized data collected from patients who had undergone a tilt-table test. Before the test, patients were interviewed using a short questionnaire regarding their presenting complaints or indications for performing a tilt-table test, their health and illness anamnesis, and any medication they used. The questionnaire is part of the tilt table test protocol performed at the outpatient clinic. The data consisted of patient age, gender, self-reported complaints, health anamnesis, daily used medications and allergies. Based on the answers provided in the questionnaire, patients were further divided into three groups: the first or control group had no known history of an orthostatic intolerance or neurodegenerative disorder, the second group had a known or suspected orthostatic intolerance disorder, and the third group had or were suspected of having a neurodegenerative disorder.

Patients who did not answer the questionnaire were excluded. Patients whose tilt-table examination results could not be obtained because of technical faults in the clinic’s tilt-table result database or because the data were corrupted with faulty readings or artifacts were excluded. Since epilepsy and its disorders can cause secondary changes in heart rate variability via ictal discharges to the autonomic nervous system, patients with known or suspected epilepsy were also excluded [12].

### 2.2. Tilt-Table Test

After completing the questionnaire, the patients were asked to lie down on the tilt table to start the examination. After the recording started, the patients’ HRV frequencies at rest were recorded for 5 min before continuing the tilt test. Afterward, the patients were rotated to a 60° tilt, and their HRV was recorded for 40 min before they were tilted back to the rest position. Some patients (*n* = 9) asked to abort the test because they developed a presyncope condition with nausea. In this research, only base (rest) HRV data were used.

After the test, patient HRV frequency data were automatically calculated using tilt-table software v2.3.20.20, and the mean averages were used.

The tilt-table software and equipment used was Task Force Monitor (Cnsystems Medizintechnik Gmbh, Graz, Austria).

The room in which the tilt-table examination occurred was air-conditioned to maintain a constant 23.0° Celsius, and it had a window without direct sunlight.

### 2.3. Statistics

Statistics calculations were conducted with IBM SPSS v26 software. Parametric tests, namely, an analysis of variance (ANOVA) and non-parametric tests, namely, Kruskal–Wallis tests, were performed. Tests for normality—Kolmogorov–Smirnov and Shapiro–Wilk tests—were conducted. A *p*-value of <0.05 was considered statistically significant. The confidence interval (CI) was set to 95%.

## 3. Results

### 3.1. General Characteristics

The final sample size consisted of 163 patients who were divided into three groups: Group 1 comprised patients with no known orthostatic intolerance or neurodegenerative disorder; this group consisted of 84 patients with a mean age of 45.8 ± 17.8 and a gender distribution of 33.3 males (%) and 66.7 females (%), as shown in Table 1.

Group 2 comprised patients with a known or suspected orthostatic intolerance; this group consisted of 50 patients with a mean age of 46.5 ± 18.6 and a gender distribution of 38 males (%) and 62 females (%), as shown in Table 1.

Group 3 comprised patients with a known or suspected neurodegenerative disease; this group consisted of 29 patients with a mean age of 55.6 ± 20.4 and a gender distribution of 34.5 males (%) and 65.5 females (%), as shown in Table 1.

The patients also had to fill in a questionnaire regarding their complaints and illness anamnesis. Group 1 patients self-reported that they had no orthostatic intolerance or neurodegenerative disorders. Patient complaints and indications for a tilt-table test were a syncope episode (*n* = 44), a presyncope episode (*n* = 20), or unclassified vertigo (*n* = 20).

Group 2 patients self-reported that they had been diagnosed with postural tachycardia syndrome (*n* = 30), orthostatic hypotension (*n* = 14), or suspected orthostatic intolerance (*n* = 6).

Group 3 patients self-reported that they had been diagnosed with Parkinson’s disease (*n* = 16), multiple sclerosis (*n* = 3), or suspected neurodegenerative disorder (*n* = 10).

### 3.2. Normalized LF and HF Powers

Normalized LF and HF powers are normalized measures derived or computed from indices that are not directly estimated from raw R-R interval data but are computed as a second step after the initial statistical estimation of the power in the LF and HF bands of the HRV spectrum. Such normalization allows for a simpler presentation of LF and HF data and comparisons with similar studies [4,13].

**Figure 1:**
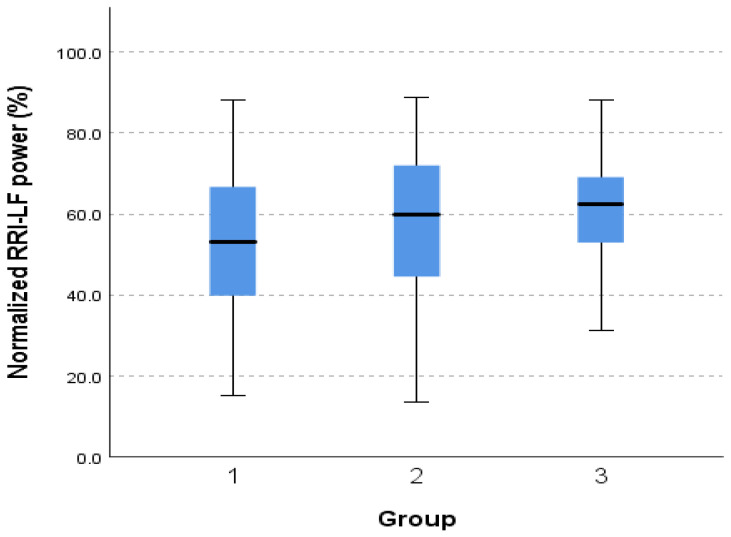
Normalized low-frequency power between patient groups.

There was a statistically significant difference in the normalized RRI-LF powers (LFnu-RRI) and RRI-HF powers (HRnu-RRI) of the patient groups. The patient group with no known orthostatic intolerance or degenerative neurological disorder (group 1) had lower RRI-LF powers, at 52.93% (SD = 18.00, *p* = < 0.05) than patients with a known or suspected orthostatic intolerance (group 2), at 58.572 (SD = 18.06, *p* = < 0.05), and patients with known or suspected neurodegenerative disorders (group 3), at 61.80% (±17.75, *p* < 0.05), as shown in Figure 1 and Table 2.

**Figure 2:**
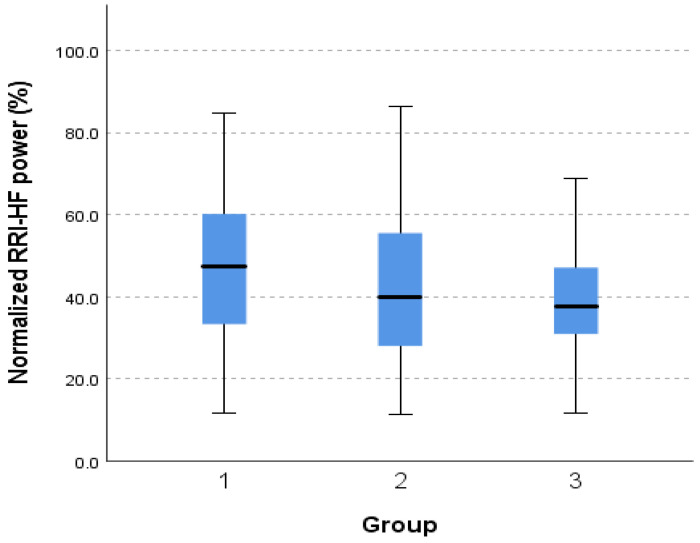
Normalized high-frequency power between patient groups.

A statistically significant difference was also found in the normalized RRI-HF power between patient groups. The patients in group 1 had increased normalized RRI-HF (HFnu-RRI) at 47.08% (SD = 17.97, *p* = < 0.05) compared with the patients in group 2 at 41.41% (SD = 18.03, *p* = < 0.05) and the patients in group 3 at 38.16% (SD = 14.70, *p* = < 0.05), as shown in Figure 2 and Table 2.

### 3.3. Frequencies

Frequencies were recorded as VLF or very-low-frequency bands (0.0033–0.04 Hz), LF or low-frequency bands (0.04–0.15 Hz), and HF or high-frequency bands (0.15–0.40 Hz). Group 1, which consisted of patients with no known orthostatic intolerance or neurodegenerative disorders, had the highest mean VLF frequency of 540.45 ms^2^ (SD: 842.65), followed by the patients in group 2 with a known or suspected orthostatic intolerance at 384.28 ms^2^ (SD: 1120.04) and the patients in group 3 with known or suspected neurodegenerative disorders at 271.55 ms^2^ (SD: 454.23). A statistically significant difference was also found in the VLF band mean results and the patient groups (*p* = 0.01).

The LF or low-frequency band was also the highest in group 1, with a mean of 531.32 ms^2^ (SD: 578.57), compared with that in group 2 at 346.2 ms^2^ (SD: 447.96) and that in group 3 at 143.21 ms^2^ (SD: 166.96). A statistically significant difference was also found in the LF band mean results and patient groups (*p* < 0.001).

The HF or high-frequency band remained the highest in group 1, with a mean of 835.87 ms^2^ (SD: 1625.42), in comparison to that in group 2 at 287.47 ms^2^ (SD: 507.15). Group 3 had a very low HF band, with a mean of 70.83 ms^2^ (SD: 75.67). A statistically significant difference was also found between the HF band mean results and patient groups (*p* =< 0.001).

The comparisons between frequency bands and patient groups are shown in Table 3.

### 3.4. LF/HF Ratio

The ratio of LF to HF power has been used to measure the sympathovagal balance, where a higher LF/HF ratio indicates a dominating sympathetic system, and a lower LF/HF ratio could indicate a dominating parasympathetic system. However, this assumption has been challenged by some authors, which will be discussed further [4,14,15].

During this study, the patients’ LF/HF-RRI ratios were also recorded, and the results are shown in Table 4. The patients in group 1 had a mean LF/HF of 1.91 (SD: 2.29), the patients in group 2 had a mean of 2.43 (SD: 2.33), and the patients in group 3 had a mean of 2.54 (SD: 2.17). A statistically significant difference was also found between the LF/HF-RRI ratios and patient groups (*p* < 0.05).

## 4. Discussion

The goal of this study was to compare the findings between patient groups. Such comparisons are difficult due to a lack of universally agreed-upon reference values. Many reasons pertain to this situation, such as both technical discrepancies and external factors, for example, gender, physical condition, comorbidities, medical drug usage, and their effects. Despite the lack of universally agreed reference values, there are studies that have attempted to present their versions of HRV norms, such as the study by F. Shafter and J.P. Ginsberg (2017) [4]. Due to the lack of universally agreed-upon norms, we compared our findings with those of other studies with similar patient demographics and methodologies.

By comparing the tilt-table results between subjectively healthy group 1 and group 2 with orthostatic intolerance, it was found that the patients in group 2 had an increased normalized LF power (*p* < 0.05), reduced absolute LF frequencies (*p* < 0.001), a reduced normalized HF power (*p* < 0.05), and a reduced absolute HF power (*p* < 0.001). The LF/HF-RII ratio was also increased (*p* < 0.05). All differences were statistically significant. According to the available data on the frequency-domain interpretation of heart rate variability, reductions in both normalized HF and absolute HF power indicate a decrease in parasympathetic nervous system modulation. The interpretation of LF power should be made more cautiously. Older studies have shown that LF power could indicate sympathetic nervous system modulation, but more recent review studies, such as one by Tam Pham et al. (2021), have described LF power to be modulated by both autonomic nervous system branches as well as baroreceptor activity [16]. During this study, data were collected from patients in the supine position, so the effect of baroreceptor modulation was minimized. LF/HF ratios have been considered to indicate sympathovagal balance. However, some authors disagree with such a statement where it has been claimed that many confounding and external factors exist that can influence LF/HF ratio results [14,15]. Therefore, LF/HF ratio interpretation should be conducted more cautiously. The patients in group 2 had an increased LF/HF ratio with a statistically significant difference (*p* < 0.05), which could indicate an imbalance in favor of the sympathetic system, but due to the available data being conflicting, such conclusions should be made cautiously. Our obtained results were compared with those of similar studies. A systemic review and meta-analysis by J. Swai et al. showed similar reductions in HF normalized and absolute powers, increased LF normalized and absolute powers, and increased LF/HF ratios [17]. A meta-analysis conducted by Fang S. et al. also showed that patients with a reduced HRV in the time and frequency domains had an increase in cardiovascular incidents [11].

The patients in group 3 with known or suspected neurodegenerative disorders exhibited changes similar to those in the patients in group 2, but the changes were much more severe. By comparing normalized HF and LF powers, it was found that the patients in group 3 had a further reduced normalized HF power than the patients in group 2 (*p* < 0.05) and a further increased normalized LF power than the patients in group 2 (*p* < 0.05). Regarding absolute powers, the patients in group 3 also had further decreased absolute LF powers (*p* < 0.001) than the patients in group 2, as well as severely decreased absolute HF powers (*p* < 0.001). Our results were compared with those of a study conducted by T. Miyagi et al. (2022). They also observed very low absolute HF powers, whereas such low values were not recorded in healthy volunteers [18]. The LF/HF ratio also increased further than in group 2. It should also be noted that the clinical onset of neurodegenerative disorders is after 50 years of age, and the mean age of group 3 was higher (55.6 ± 20.4) than that of group 1 (45.8 ± 17.8) and group 2 (46.5 ± 20.04) [19,20,21]. A study conducted by J Am Heart Assoc showed that heart rate variability can be reduced by normal physiological causes due to aging [21]. Another factor that can influence the LF/HF ratio is patient activity levels. Patients with neurodegenerative disorders tend to be less active than younger, healthier patients. A study conducted by C. Ashok et al. showed that active people who visit the gym had a lower LF/HF ratio than people who had a more sedentary lifestyle [22]. Another study by AV Vinay et al. showed that yoga can reduce the LF/HF ratio [23]. Thus, when we interpreted the lower absolute HF and LF values, we could not rule out such factors.

We investigated the results of the LF/HF ratios further. As previously mentioned, there are no universally agreed upon LF/HF ratio reference values, but some studies, such as one by Yilmaz M. et al., have suggested a normal range of 1.5–2.0. [24] Although group 1 was within this normal range, groups 2 and 3 were not. A study by Nunan et al. suggested an LF/HF median norm of 2.8 [25]. By comparing our results, it was found that all groups were below this norm (1.91, 2.43, and 2.54, respectively). To investigate further reasons for the increased LF/HF, we analyzed studies that also reported a higher LF/HF ratio. A study by L. Weivei showed an increase in LF/HF ratios due to indoor environment temperature changes. It is possible that, during our study, the environment had an effect, as our study was conducted in an outpatient clinic during both colder winter and warmer summer months, where patients experienced rapid temperature changes when entering the clinic [26]. A study by N. Sadig showed an increase in LF/HF ratios due to psychoemotional stress caused by medical students. Before undergoing the tilt-table examination, patients were warned as part of informed consent that the test could provoke a feeling of vertigo or presyncope. Possible patient anxiety of a repeat of experienced symptoms is also a valid possibility [27].

Another important factor that could affect the LF/HV ratio is patient medicine usage. A study conducted by E. Gernot found that certain medicine groups can affect HRV, such as ACE inhibitors, which can increase absolute HF and LF powers, while antidepressants can reduce all frequency domain parameters [28]. While the patient questionnaire did include a section regarding medicine usage, most patients responded that they either do not remember or the answers where too vague such as “heart drops” for further investigation.

VLF absolute power was also recorded, and it showed statistically significant differences (*p* = 0.01) between groups 1, 2, and 3. However, according to the available data, VLF correlates with body temperature regulation and vasomotor activity [4,5,9]. It is possible to record VLF absolute power in a 5-min interval, but most studies support a longer recording of 24 h [4]. While VLF powers have been studied as an important clinical prognosis factor for patients with cardiovascular disease, our study findings were not investigated further due to them being recorded for only 5 min and, thus, having a limited investigative value [29].

Taking into account the available theoretical data and the data obtained from this study, it was determined that the patients in groups 2 and 3 had reduced parasympathetic modulation and, thus, parasympathetic activity. However, an increase in LF power does not indicate increased sympathetic activity. The results also show that group 3 had a much more severe reduction in parasympathetic activity than group 2. However, age can also play an influencing factor in reducing HRV, and group 3 showed the most reduced HRV but also the highest patient mean age. The interpretation of our results was also made difficult by the possible influence of both external factors and internal factors, which could have affected the findings.

The main limitations of this study were as follows: due to a lack of objective medical records, patient grouping was based on patient self-reported data. With available patient medical records, patient division into groups could be more precise. Additionally, this study was conducted in an outpatient setting. Although the patients were educated about proper preparation before the test, external factors could have affected the results, such as physical exertion before arriving at the clinic, which could not be completely ruled out. Another important factor that had the potential of skewing the HRV results was patient medicine usage. The studies available show that multiple medicine groups have the potential to both increase or decrease HRV. While the questionnaire did include questions regarding medicine usage, patient answers were too vague to offer any evaluation. This retrospective study was conducted without a healthy volunteer control group. Considering the limitations encountered during the study, we believe that this study could be repeated in an in-patient or hospitalized patient setting, using a prospective design. Conducting an HRV examination in a hospitalized setting and different clinics would allow the recruitment of healthy volunteers for a healthy control group, a larger patient size, the availability of more precise and detailed patient medical history, and information regarding patient medicine usage. Early in the studies, we encountered some technical errors which made some patient data unavailable in the databases, potential for selection bias, and difficulties controlling all variables. A prospective study could have limited such limitations. Additionally, a 24 h HRV monitoring could provide more detailed and accurate results. The study also referenced many other studies regarding possible cardiovascular events. To further investigate possible cardiac events, patient follow-up examination after a 1 year could lead to more significant results. Taking into account the statistically significant data obtained in this retrospective study, which showed a significant tendency in HRV for patients with orthostatic intolerance and patients with neurodegenerative disorders, a prospective study with a control group could be performed to confirm the tendency and trend observed in this study.

### Clinical Implications

Our study has found that patients with neurodegenerative disorders have more severely reduced HRV than patients with orthostatic intolerance. This could be clinically significant in patient screening where abnormally low HRV could indicate a possible neurodegenerative disorder that would require further neurological examination. For patients with known neurodegenerative disorders HRV examination could help in monitoring their cardiovascular health and prompt prophylaxis.

Patients with orthostatic intolerance HRV examination can be used to monitor their cardiovascular health and response to treatment. This study has also shown a tendency for HRV to be reduced more severely in patients with neurodegenerative disorders. HRV could be potentially used as a dynamic marker to evaluate patients with neurodegenerative disorders their illness stage and progression.

During the study, we found that the differences in normalized (LFnu and HFnu) powers between the groups, while statistically significant, there was still a serious range overlap. This could indicate HRV differences between patients with less severe clinical symptoms. HRV differences are also less pronounced. However, the possibility of mistakes in patient grouping could also lead to such results since patients were grouped based on their self-reported health anamnesis. A more precise patient grouping could lead to more precise results.

## 5. Conclusions

Our study analyzed a sample of 163 patients who underwent a tilt-table test in an outpatient clinic. The tests showed that patients with a known or suspected orthostatic intolerance had reduced HRV. A frequency-domain analysis showed reduced parasympathetic activity, which, in turn, led to reduced heart rate variability. According to available studies, it is possible that such patients are at an increased risk of cardiogenic incidents, such as arrhythmia and cardiogenic shock. In patients with known or suspected neurodegenerative disorders, parasympathetic activity was much more severely reduced, which, according to other studies, could indicate an even higher risk of cardiogenic incidents. Therefore, patients with a neurodegenerative disorder should be examined more thoroughly for potential cardiological issues. HRV analysis also has the potential to be used as a dynamic clinical marker to evaluate patients with neurodegenerative disorders and their illness progression and stage.

Despite the results and values obtained from HRV examinations via the frequency domain in this study, a lack of standardization regarding reference values and methodology remains a major obstacle in introducing HRV examinations into wider clinical practice. Additionally, it is possible that both external factors and internal factors, which are difficult to control, have an effect on heart rate variability examinations. Further studies in this area are required.

## Figures and Tables

**Table 1:** Patient general demographics after grouping.

		Group 1 ^1^ *n* = 84	Group 2 ^2^ *n* = 50	Group 3 ^3^ *n* = 29
Gender	Male (%)	33.3	38	34.5
	Female (%)	66.7	62	65.5
Mean age (years)		45.8 ± 17.8	46.5 ± 18.6	55.6 ± 20.4

^1^ Patients with no known orthostatic intolerance or neurodegenerative disorders. ^2^ Patients with known or suspected orthostatic intolerance. ^3^ Patients with known or suspected neurodegenerative disorders.

**Table 2:** Comparison of LF and HF normalized powers between groups.

	Group 1 ^1^	Group 2 ^2^	Group 3 ^3^	*p* Value
LFnu-RRI % (SD)	52.93 (18.00)	58.57 (18.06)	61.80 (14.74)	<0.05
HFnu-RRI % (SD)	47.08 (17.97)	41.41 (18.03)	38.16 (14.70)	<0.05

^1^ Patients with no known orthostatic intolerance or neurodegenerative disorders. ^2^ Patients with a known or suspected orthostatic intolerance. ^3^ Patients with known or suspected neurodegenerative disorders.

**Table 3:** Means of frequency powers VLF-RRI, LF-RRI, and HF-RRI and comparisons between groups.

Frequency Power	Group 1 ^1^	Group 2 ^2^	Group 3 ^3^	*p* Value
VLF-RRI ms^2^ (SD)	540.45 (842,65)	384.28 (1120.04)	271.55 (454.23)	=0.01
LF-RRI ms^2^ (SD)	531.32 (578.57)	346.20 (447.96)	143.21 (166.96)	<0.001
HF-RRI ms^2^ (SD)	835.87 (1625.42)	297.46 (507.15)	70.83 (75.67)	<0.001

^1^ Patients with no known orthostatic intolerance or neurodegenerative disorders. ^2^ Patients with a known or suspected orthostatic intolerance. ^3^ Patients with known or suspected neurodegenerative disorders.

**Table 4:** LF/HF ratios comparison between groups.

	Group 1 ^1^	Group 2 ^2^	Group 3 ^3^	*p* Value
LF/HF-RRI ratio (SD)	1.91 (2.29)	2.43 (2.33)	2.54 (2.17)	<0.05

^1^ Patients with no known orthostatic or neurodegenerative disorders. ^2^ Patients with a known or suspected orthostatic intolerance. ^3^ Patients with known or suspected neurodegenerative disorders.

## Data Availability

Data are available upon request due to ethical restrictions. All the data included in this study are available upon request from the corresponding author. The data are not publicly available and are stored in the patient tilt-table result database located in the polyclinic “VCA Pļavnieki” in Riga, Latvia.
